# Female-biased sexual dimorphism of corticotropin-releasing factor neurons in the bed nucleus of the stria terminalis

**DOI:** 10.1186/s13293-019-0221-2

**Published:** 2019-01-28

**Authors:** Katsuya Uchida, Hiroko Otsuka, Masahiro Morishita, Shinji Tsukahara, Tatsuya Sato, Kenji Sakimura, Keiichi Itoi

**Affiliations:** 1 Laboratory of Information Biology, Graduate School of Information Sciences, Tohoku University, Sendai City, Japan; 2 Department of Regulation Biology, Graduate School of Science and Engineering, Saitama University, Saitama City, Japan; 3 Department of Cellular Neurobiology, Brain Research Institute, Niigata University, Niigata City, Japan

**Keywords:** Mouse, Stereology, Immunofluorescence, Gonadectomy, Affection

## Abstract

**Background:**

The bed nucleus of the stria terminalis (BNST) contains the highest density of corticotropin-releasing factor (CRF)-producing neurons in the brain. CRF-immunoreactive neurons show a female-biased sexual dimorphism in the dorsolateral BNST in the rat. Since CRF neurons cannot be immunostained clearly with available CRF antibodies in the mouse, we used a mouse line, in which modified yellow fluorescent protein (Venus) was inserted to the CRF gene, and the Neo cassette was removed, to examine the morphological characteristics of CRF neurons in the dorsolateral BNST. Developmental changes of CRF neurons were examined from postnatal stages to adulthood. Gonadectomy (GDX) was carried out in adult male and female mice to examine the effects of sex steroids on the number of CRF neurons in the dorsolateral BNST.

**Methods:**

The number of Venus-expressing neurons, stained by immunofluorescence, was compared between male and female mice over the course of development. GDX was carried out in adult mice. Immunohistochemistry, in combination with Nissl staining, was carried out, and the effects of sex or gonadal steroids were examined by estimating the number of Venus-expressing neurons, as well as the total number of neurons or glial cells, in each BNST subnucleus, using a stereological method.

**Results:**

Most Venus-expressing neurons co-expressed *Crf* mRNA in the dorsolateral BNST. They constitute a group of neurons without calbindin immunoreactivity, which makes a contrast to the principal nucleus of the BNST that is characterized by calbindin immunostaining. In the dorsolateral BNST, the number of Venus-expressing neurons increased across developmental stages until adulthood. Sexual difference in the number of Venus-expressing neurons was not evident by postnatal day 5. In adulthood, however, there was a significant female predominance in the number of Venus expressing neurons in two subnuclei of the dorsolateral BNST, i.e., the oval nucleus of the BNST (ovBNST) and the anterolateral BNST (alBNST). The number of Venus-expressing neurons was smaller significantly in ovariectomized females compared with proestrous females in either ovBNST or alBNST, and greater significantly in orchiectomized males compared with gonadally intact males in ovBNST. The total number of neurons was also greater significantly in females than in males in ovBNST and alBNST, but it was not affected by GDX.

**Conclusion:**

Venus-expressing CRF neurons showed female-biased sexual dimorphism in ovBNST and alBNST of the mouse. Expression of Venus in these subnuclei was controlled by gonadal steroids.

## Background

Corticotropin-releasing factor (CRF) neurons, in the paraventricular nucleus of the hypothalamus (PVH), play a pivotal role in the regulation of hypothalamic-pituitary-adrenal axis and control synthesis of glucocorticoid that is essential for protecting an organism from stress [1]. CRF neurons are also distributed in the brain regions outside the PVH such as the cerebral cortex [2], the inferior olivary nucleus [3], Barrington’s nucleus [4], the central nucleus of the amygdala (CeA) [5], and the bed nucleus of the stria terminalis (BNST) [6]. In the rat, CRF neurons in the brain are readily detectable by immunohistochemistry, and the staining becomes more intense after colchicine injection into the brain ventricle to block an axonal flow. However, CRF neurons cannot be immunostained clearly with any available CRF antibody in most brain regions in the mouse, even with colchicine treatment [7, 8]. We could overcome this difficulty by using a mouse line, in which modified yellow fluorescent protein (Venus) was inserted into the CRF genomic locus (CRF-Venus) [8]. Subsequently, another mouse line (CRF-Venus∆Neo) was generated by deletion of the pgk-1 promoter-driven neomycin phosphotransferase gene (Neo cassette) from the CRF-Venus genome, and the Venus expression was profoundly enhanced in the CRF-VenusΔNeo compared with the CRF-Venus [8, 9]. Recently, distribution of CRF neurons was unraveled throughout the mouse brain by staining Venus, in the CRF-VenusΔNeo, with anti-green fluorescent protein (GFP) antibody that cross-reacts with Venus [8, 9].

The BNST is one of the brain regions that contain the highest density of CRF neurons in the mouse [9] and the rat [6, 10]. It is primarily divided into anterior and posterior divisions, and CRF neurons are reported to be distributed in the former in the rat. CRF neurons are also reported to be expressed most prominently in the oval nucleus of the BNST (ovBNST), among other subnuclei in the anterior division of BNST, in the rat [6, 10], which is located in the central portion of the dorsolateral BNST.

The BNST is structurally a continuum of the amygdaloid nucleus and referred to as the “extended amygdala.” The basolateral amygdala and two downstream structures, the CeA and the BNST, are implicated in anxiety and fear [11, 12]. The CeA was proposed to play a role in short-duration fear responses, whereas the BNST in sustained responses, in experimental animals [11, 12]. By functional magnetic resonance imaging studies in humans, BNST activation was related to the anticipation of threat [13].

It was reported by Fukushima and colleagues that the number of CRF neurons in the dorsolateral BNST of the rat is greater in females than in males [14]. Interestingly, the number of CRF neurons in dorsolateral BNST of female rats decreased by treatment with testosterone during the neonatal period [15]. Therefore, the levels of gonadal steroids in a developmentally critical period may determine the fate of the BNST neurons under development that is committed to become CRF-expressing neurons in adulthood.

Although the structure and function of the BNST have been studied extensively in the rat, the use of mice is becoming much more important for the functional analysis of a particular neuronal population in the brain. Mice can be genetically manipulated, and genetically engineered mice are becoming indispensable tools for both anatomical and physiological studies of the brain. With the use of a diver mouse line, in combination with viral vectors, pathway tracing of a particular class of neurons is possible [16]. Opto- or chemogenetic experiments have enabled us to target selective neurons in the mouse brain and control them with high temporal and spatial precision [17]. The present study was aimed at elucidating the morphological characteristics of CRF neurons in the dorsolateral BNST of mice, paying particular attention to its sexual difference and the effects of gonadal steroids, and the CRF-Venus∆Neo mouse was employed to this end. First, the changes in the BNST morphology were observed across postnatal stages until adulthood to elucidate the time course of development of the sexual dimorphism of CRF neurons. Second, gonadectomy (GDX) was carried out in adult male and female mice to examine the effects of sex steroids on the morphology of BNST CRF neurons. The present study may provide a structural basis for studying the role of BNST in sex-dependent emotional behaviors in the mouse.

## Methods

### Animals

All animal procedures were approved by the Committee on Use and Care of Animals, Tohoku University. The CRF-Venus∆Neo mice were used [8, 9]. The CRF-Venus mouse was generated in our laboratory by inserting the Venus gene into the CRF gene by homologous recombination [8]. However, Venus-expressing neurons did not always co-express CRF in the CRF-Venus mouse [8]. We then generated the CRF-Venus∆Neo by deleting the Neo cassette from the genome of the CRF-Venus mouse. In the CRF-Venus∆Neo mouse, the intensity of Venus expression is profoundly increased compared with the CRF-Venus mouse, and most Venus-expressing neurons co-express CRF mRNA not only in the PVH but also in other brain regions, including the BNST [9]. The animals were allowed free access to chews (Labo MR Stock, NOSAN Co., Yokohama, Japan) and water and were maintained on a 12-h light/dark cycle (lights on 07:00–19:00) at a constant temperature of 23 °C and 40–50% humidity. For observation of Venus-expressing neurons of the BNST over the course of development, postnatal day 2 (P2) (*n* = 5), P5 (*n* = 7), and 3-month-old male and female mice (*n* = 4) were used in histological experiments. P2 was chosen as the pre-apoptotic stage and P5 as the peak of the apoptotic stage of development, according to the study by Gotsiridze and colleagues [18]. GDX was carried out on 3-month-old male (*n *= 7) and female mice (*n* = 8) under anesthesia with a combination of 3 μg medetomidine, 40 μg midazolam, and 50 μg butorphanol per 10 g body weight. Orchiectomy (ORX) was carried out by the inguinal approach. The testicle was exposed by pulling the vas deferens through the skin incision. After the vas deferens was ligated and severed, the testicle was removed. The contralateral testicle was removed in the same manner. Ovariectomy (OVX) was carried out by the dorsolateral approach. After identifying the ovary in the retroperitoneal adipose tissue, a suture was made around the distal uterine horn, and the ovary was removed. The contralateral ovary was removed in the same manner. Male and female mice for the gonadally intact groups (*n* = 8 for each group) underwent sham surgery. After surgery, 2–3 weeks were allowed for recovery, and these mice were used for the morphometrical experiments. For identifying the proestrous stage, vaginal smears were taken for 14 consecutive days, and the estrous cycles were determined [19].

### Tissue preparation, immunofluorescence, and immunohistochemistry

Mice were deeply anesthetized with chloral hydrate (400 mg/kg body weight, intraperitoneally) and perfused with 0.9% saline followed by 4% paraformaldehyde (PFA) in 0.1 M phosphate buffer (PB, pH 7.4). The brain was post-fixed overnight in 4% PFA and stored in 30% sucrose in 10 mM PB (pH 7.4) at 4 °C for 48 h. Thirty-micrometer-thick sections were serially cut on a cryostat, and the free-floating sections were rinsed in 10 mM phosphate-buffered saline (PBS) (pH 7.4). For the immunofluorescence, the sections were incubated for 30 min with 1% normal donkey or goat serum diluted in PBS containing 0.1% (*w*/*v*) Triton X-100 (PBS-T) after being washed with PBS-T. The sections were then incubated overnight at 4 °C with rabbit anti-GFP (1:2000, Dr. Masahiko Watanabe, Hokkaido University) [20] and/or goat anti-calbindin (as a marker for the principal nucleus, 1:500; Santa Cruz Biotechnology, Dallas, TX, USA). After being washed with PBS-T, the sections were incubated with Alexa 488-conjugated donkey anti-rabbit IgG (1:250; Thermo Fisher Scientific, Waltham, MA, USA) and Alexa 594-conjugated donkey anti-goat IgG (1:1000; Thermo Fisher Scientific) for 2 h at room temperature and then washed with PBS-T. The sections were mounted with Aqua-Poly/Mount (Polysciences, Inc., Warrington, PA, USA) and imaged using a CCD camera (Pixera 600CL, Santa Clara, CA, USA) under a fluorescent microscope (DMR, Leica, Wetzlar, Germany), and the number of cells was counted. We counted the number of Venus-expressing neurons on each side of the respective BNST region.

Immunohistochemistry was carried out to estimate the volume of the nucleus and the number of cells in it: the sections were incubated overnight at 4 °C with rabbit anti-GFP (1:2000). After being washed with PBS-T, the sections were treated with 1% H_2_O_2_ to inactivate endogenous peroxidases. After another wash with PBS-T, the sections were incubated with biotinylated goat anti-rabbit IgG (1:1000; Vector Laboratories, Burlingame, CA, USA) for 2 h at room temperature, rinsed with PBS-T, and incubated with avidin-biotin complex (ABC; Vector Laboratories) for 1 h at room temperature. The staining was then visualized by incubating the sections in 0.05% diaminobenzidine and 0.02% H_2_O_2_ in PBS. The sections were spread onto amino silane-coated microscope slides, and then, Nissl staining was carried out with thionin solution.

### Fluorescence in situ hybridization combined with immunofluorescence

Digoxigenin (DIG)-labeled cRNA probes were synthesized in vitro by transcribing the pGEM-7zf vector (Promega, Madison, WI, USA) encoding a mouse *Crf* fragment (nucleotides 138–958; GenBank accession number, BC119036; Dr. Shuhei Horio, Tokushima University, Japan). The sections were hybridized and washed stringently, as was described elsewhere [20]. For immunofluorescence detection of DIG, the sections were blocked for 30 min with DIG blocking solution: Tris-HCl buffer (pH 7.4) containing 1% blocking reagent (Roche Diagnostics, Basel, Switzerland) and 4% normal sheep serum. The sections were blocked again with 0.5% TSA blocking reagent (PerkinElmer) in TNT buffer for 30 min. The sections were then incubated for 1 h with peroxidase-conjugated anti-DIG (1:1000; Roche Diagnostics) and the Cy3-TSA plus amplification kit (PerkinElmer). For immunofluorescence of Venus, the sections were blocked for 30 min with normal goat serum. The sections were incubated with rabbit anti-GFP antibody (1:2000, Dr. Watanabe) overnight at 4 °C. After being washed with PBS-T, the sections were incubated with Alexa 488-conjugated donkey anti-rabbit IgG (1:250; Thermo Fisher Scientific) for 2 h at room temperature and then washed with PBS-T. The sections that were stained dually with fluorescence in situ hybridization and immunofluorescence were mounted with Aqua-Poly/Mount (Polysciences, Inc.) and imaged by a confocal laser-scanning microscope (TCS SPE, Leica).

### Morphometrical analysis by stereology

In another set of experiments, stereology was used to estimate the number of Venus-expressing neurons and the total number of neurons or glial cells in ovBNST and alBNST, as well as the volume of each subnucleus. These parameters were examined in gonadally intact males (Sham-orchiectomized), orchiectomized males, proestrous females (Shan-ovarictomized), or ovariectomized females. Thirty-μm sections were collected every 90 μm from 0.38 to 0.02 mm posterior to the bregma [21], and they were co-stained with anti-GFP antibody and thionin. After staining the Venus-expressing neurons with DAB, the sections were washed with distilled water, and then the sections were incubated with 0.5% thionin solution (pH 4.5) for 10 min. After washing with distilled water, the sections were dehydrated in alcohol series. The sections were finally mounted with Entellan New (Merck Millipore, Burlington, MA, USA). Stereological analysis was carried out using Stereo Investigator software (MBF Bioscience Inc., Williston, VT, USA). We used the optical fractionator method according to the system workflow of Stereo Investigator. We first traced the outlines of ovBNST and alBNST on the left side of each section to calculate the volume of the selected area and the number of cells within the area. We defined each subnucleus in the BNST in thionin-stained sections according to Ju and colleagues [10, 22]. Each subnucleus could be discriminated from the other without difficulty in reference to the cytoarchitectonic and morphological characteristics of the cells [10, 22]; whereas ovBNST neurons were tightly packed, and their nuclei were round-shaped, alBNST neurons were small-sized and distributed sparsely. Cells could be defined as neurons by the densely thionin-stained cytoplasm, containing an oval- or spherical-shaped nucleus with a clear nucleolus. The nucleus of a glial cell was smaller and stained densely with thionin, while its cytoplasm was barely stained [23]. Cells were excluded from either category when they were located near the capillaries and could potentially be endothelial cells.

### Statistical analysis

For all statistical analyses, we used GraphPad Prism 7 software (GraphPad Software, Inc., La Jolla, CA, USA). Student’s *t*-test was used for analyzing the differences in the number of Venus-expressing neurons between adult male and female mice in the dorsolateral-, anterodorsal-, and ventral BNST. Two-way analysis of variance (ANOVA) was used to determine the effects of age and sex on the number of Venus-expressing neurons across developmental stages. Two-way ANOVA was also used to determine the effects of sex and GDX on the number of Venus-expressing neurons, the total number of neurons or glial cells, and the volume of the subnucleus in either ovBNST or alBNST, as well as to determine the effects of sex and location (subnucleus) on the number of Venus-expressing neurons in either intact or gonadectomized mice. If there was a significant interaction between the factors, Tukey’s multiple comparisons test was carried out as a *post-hoc* analysis. All data are shown as the mean ± SEM. Statistical differences were considered significant at *p* < 0.05.

## Results

### Distribution of Venus-expressing neurons in the BNST

**Fig. 1:**
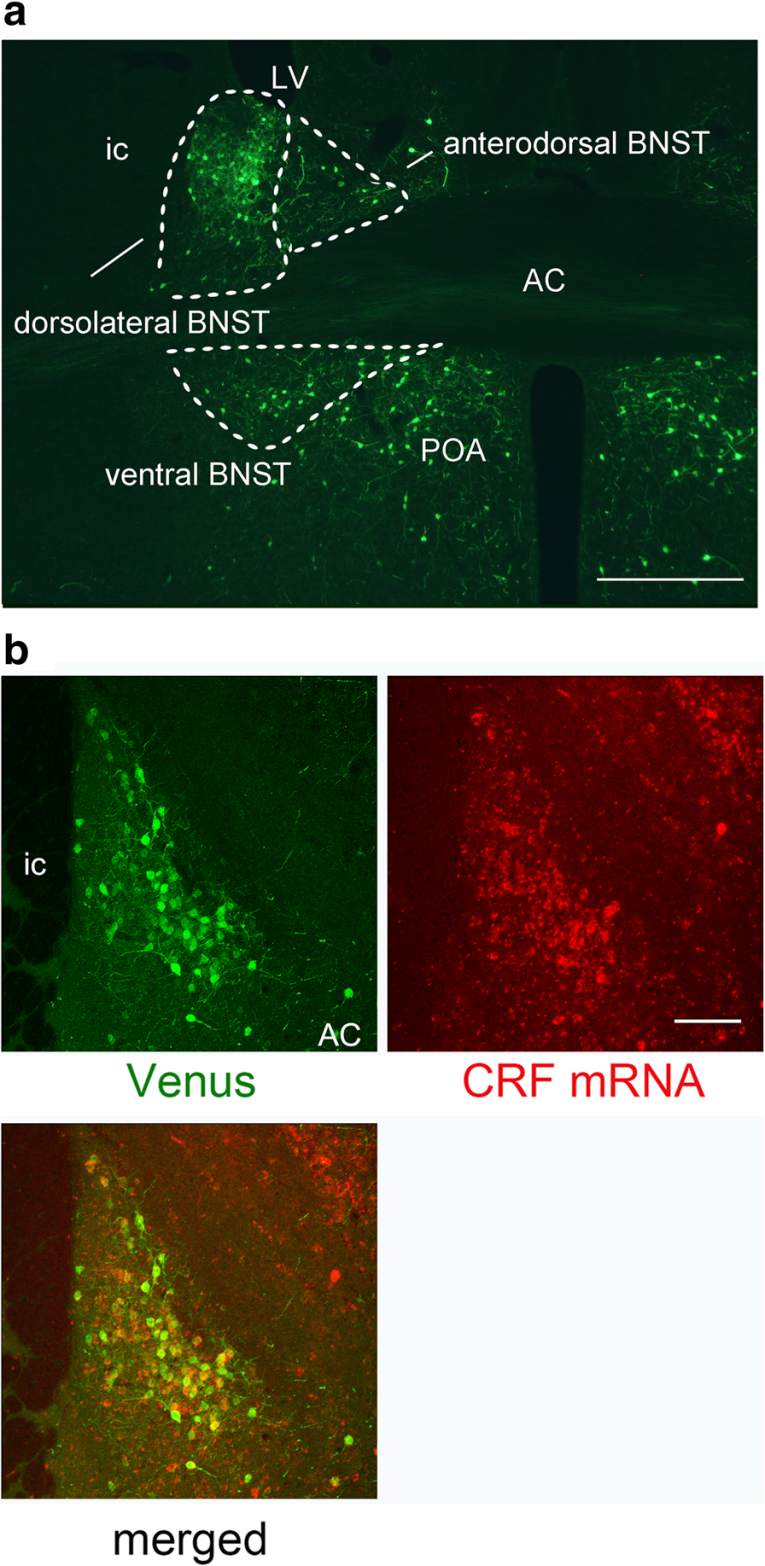
Distribution of Venus-expressing neurons in the anterior division of the BNST and co-localization of Venus immunofluorescence with *Crf* mRNA in the dorsolateral BNST. **a** Photomicrograph showing the Venus-expressing neurons in the anterior division of the BNST. White dashed lines indicate the boundaries of the subnuclei in the anterior BNST. LV, lateral ventricle; ovBNST, oval nucleus of the BNST; alBNST, anterolateral BNST; AC, anterior commissure; POA; anterior preoptic area; ic, internal capsule. Scale bar = 200 µm. **b** Confocal images showing Venus (immunofluorescence, upper left panel) and *Crf* (fluorescence in situ hybridization, upper right panel) in the dorsolateral BNST. The Venus and *Crf* are shown as green and red, respectively. The lower left panel shows a merged image of Venus and *Crf* mRNA. Scale bar = 100 μm

To elucidate the localization of CRF neurons in the BNST, we carried out immunofluorescence and examined the distribution of Venus-expressing neurons using the CRF-Venus∆Neo mice. Venus-expressing neurons were distributed broadly in the anterior division of the BNST that includes the dorsolateral BNST, the anterodorsal BNST, and the ventral BNST (Fig. 1a). It was revealed by confocal microscopy that most Venus-expressing neurons were co-localized with *Crf* mRNA in the BNST. As was shown in Fig. 1b, for example, almost all Venus-expressing neurons co-expressed *Crf* mRNA in the dorsolateral BNST. Although a small number of *Crf *mRNA-positive neurons were devoid of Venus, a great majority of *Crf* mRNA-positive neurons co-expressed Venus. Therefore, Venus-expressing neurons were considered to represent CRF-producing neurons.

**Fig. 2:**
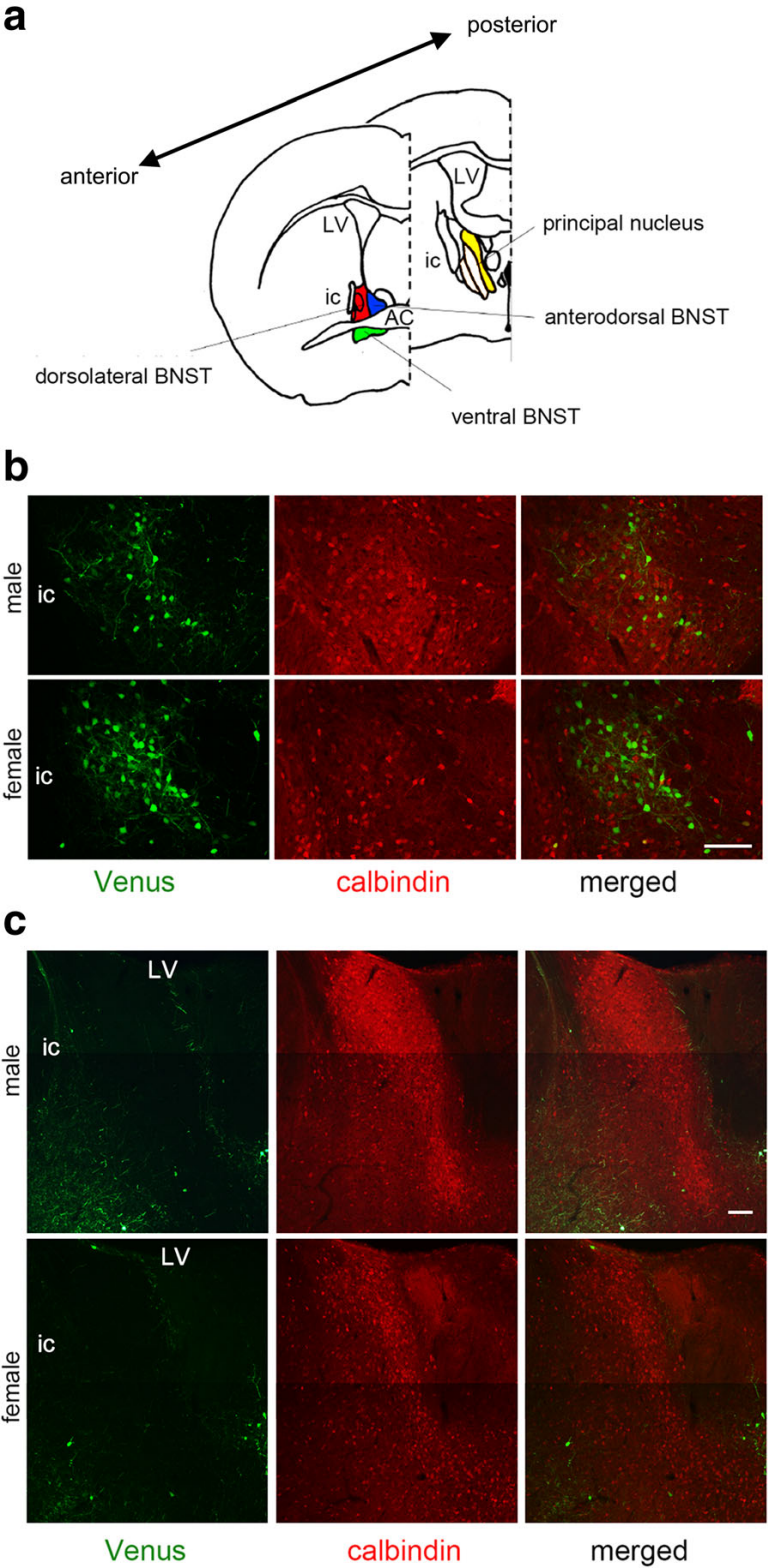
Differential distribution of Venus-expressing neurons in dorsolateral BNST and calbindin-positive neurons in the principal nucleus of the BNST in the mouse. **a** A diagram illustrating the cytoarchitecture of the anterior and posterior divisions of the BNST in coronal sections. LV, lateral ventricle; ic, internal capsule; AC, anterior commissure. The area in red shows the dorsolateral BNST, blue the anterodorsal BNST, green the ventral division of the BNST, and yellow the principal nucleus of the BNST. **b** Photomicrographs showing double immunofluorescence for Venus-expressing neurons (green) and calbindin-positive neurons (red) in the dorsolateral BNST. **c** Photomicrographs showing Venus-expressing neurons (green) and calbindin-positive neurons (red) in the principal nucleus. The right-most panels show the merged images of Venus-expressing and calbindin-positive neurons. Scale bar = 100 μm

Topographical localization of the Venus-expressing neurons was examined with the double immunofluorescence of Venus and calbindin D-28k and the immunohistochemistry of Venus in combination with Nissl staining. Calbindin D-28k is a calcium-binding protein utilized as a marker for the gamma-aminobutyric acid (GABA)-containing interneurons, but it is also a marker for the principal nucleus of the BNST [24]. To examine whether Venus-expressing (CRF) neurons all belong to a distinctive group of neurons in the dorsolateral BNST, or whether part of them belong to the principal nucleus, double immunofluorescence was carried out with anti GFP- and anti-calbindin antibody from the anterior to the posterior division of the BNST (Fig. 2b, c). Another purpose of this study was to examine whether CRF neurons in the dorsolateral BNST that are known to contain GABA [9] also express calbindin or not. As shown in Fig. 2b, a cluster of Venus-expressing neurons was observed clearly in the dorsolateral BNST. Remarkably Venus-expressing neurons in females outnumbered those in males in the dorsolateral BNST (Fig. 2b). A small number of calbindin-immunoreactive neurons could be observed in the same section, but this group of cells was not a continuum of a cluster of calbindin-positive neurons in the principal nucleus. Venus-expressing neurons never co-expressed calbindin immunoreactivity in the dorsolateral BNST (Fig. 2b). The principal nucleus could be identified clearly by calbindin immunostaining, and the number of calbindin-positive neurons was much greater in males than in females in this nucleus (Fig. 2c). However, very few Venus-expressing neurons were observed in this region, and they never co-localized with calbindin (Fig. 2c). Thus, the CRF neurons in the dorsolateral BNST belong to a distinctive group of neurons which is completely different from the principal nucleus.

**Fig. 3:**
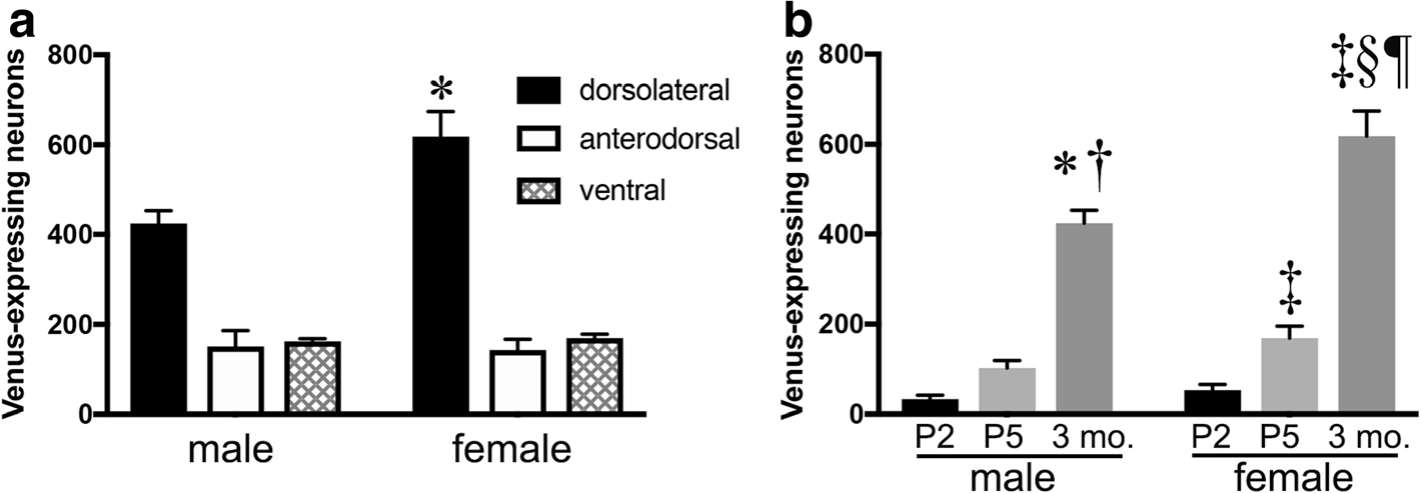
Sex differences in the number of Venus-expressing neurons in the subnuclei of the anterior division of BNST. **a** The number of Venus-expressing neurons in adult male and female mice. *p < 0.0001 vs. male in the dorsolateral BNST. **b** The changes in the number of Venus-expressing neurons in the dorsolateral BNST from early postnatal stages to adulthood. Two-way ANOVA indicated the main effect of sex or age on the number of Venus-expressing neurons in the dorsolateral BNST. *p < 0.001 vs. P2 male mice; ^†^*p* < 0.001 vs. P5 male mice; ^‡^*p* < 0.0001 vs. P2 female mice; ^§^*p* < 0.001 vs. P5 female mice; ^¶^*p* < 0.01 vs. 3 month-old male mice (the data of adult male mice in **b** are the same as the dorsolateral data in **a**. Data are shown as the means ± SEM

To confirm the female-biased sexual dimorphism in the number of CRF neurons in the dorsolateral BNST (*vide supra*), and to examine whether the female predominance is confined to the area, we counted the number of Venus-expressing neurons in the dorsolateral-, anterodorsal-, and ventral BNST in both sexes in adulthood (at 3 months of age). There was approximately 1.5-fold as many Venus-expressing neurons in females as in males in dorsolateral BNST (female 613 ± 55 cells vs. male 423 ± 28 cells, *p* < 0.05; Fig. 3a). However, there were no significant differences in the number of Venus-positive neurons between males and females in the anterodorsal and ventral BNST (Fig. 3a). Therefore, the dorsolateral BNST is the only region that showed a female-biased sex difference.

We sought to examine at what developmental stage the female-biased sex difference becomes evident in the dorsolateral BNST. The BNST neurons undergo apoptosis during the developmental course, so we examined the mice at P2 (before the peak of apoptosis) and P5 (at the peak of apoptosis), as compared with those at 3 months of age. The number of Venus-expressing neurons increased over the course of development, from P2 to 3 months, but there was no significant sex difference from P2 to P5 (Fig. 3b). The number of Venus-expressing neurons increased dramatically thereafter, and the female predominance became evident in adulthood (3 months of age) (Fig. 3b). Two-way ANOVA indicated a significant interaction between sex and age (*F* (2, 26) = 4.903, *p* < 0.05), as well as the main effects of sex (*F* (1, 26) = 18.2, *p* < 0.001) and age (*F* (2, 26) = 154.6, *p* < 0.0001). Tukey’s multiple comparisons test indicated that the number of Venus-expressing neurons was significantly greater in 3-month-old male- vs. P2 (*p* < 0.001) and P5 male mice (*p* < 0.001) and significantly greater in 3-month-old female- vs. P2 (*p* < 0.0001) and P5 female mice (*p* < 0.001) (Fig. 3b). The number of Venus-expressing neurons was significantly greater in 3-month-old female vs. 3-month-old male (*p* < 0.01) (Fig. 3b).

### Effects of gonadal steroids on the number of Venus-expressing neurons in the dorsolateral BNST

**Fig. 4:**
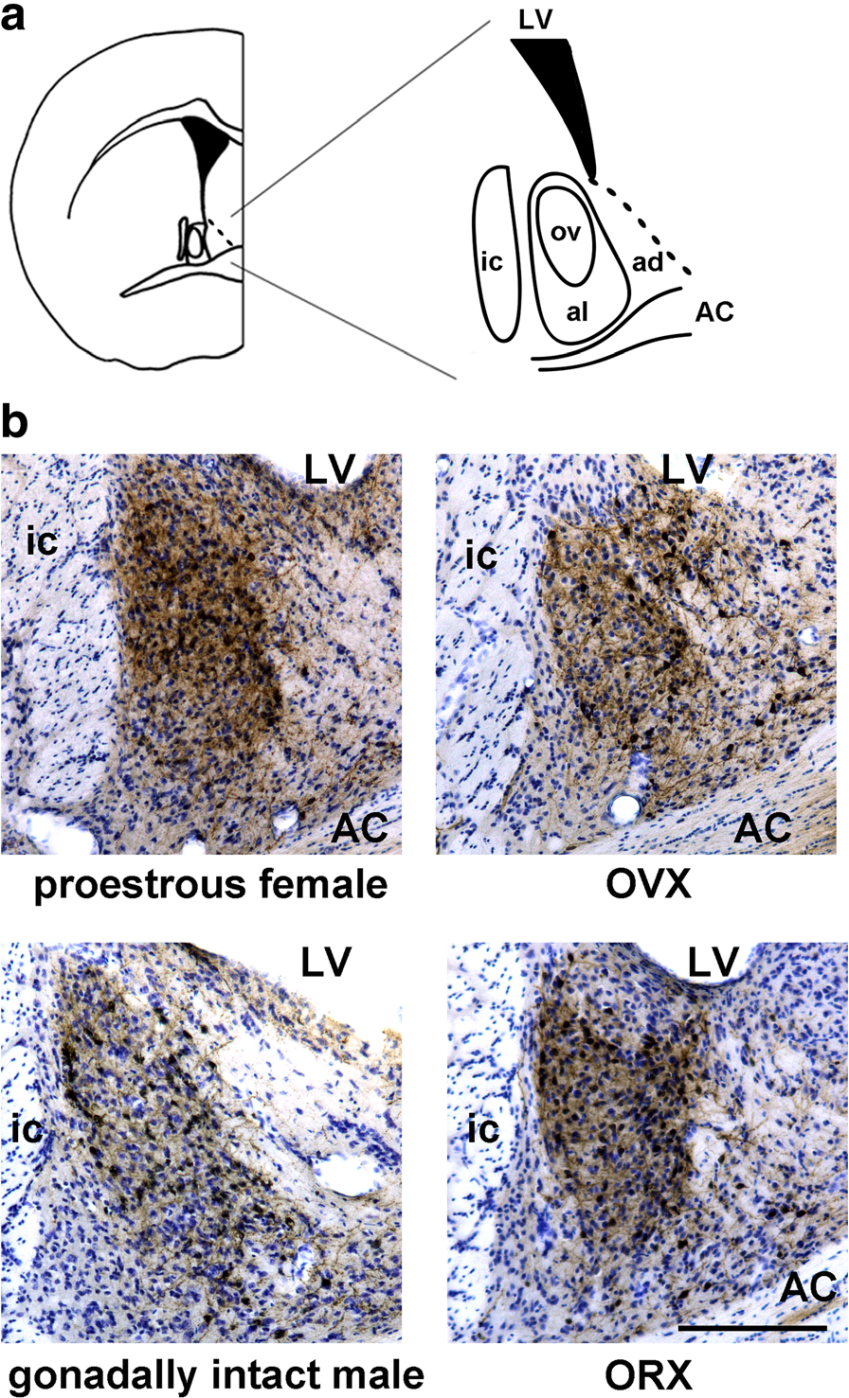
The effect of gonadectomy on the morphology of Venus-expressing neurons in the dorsolateral BNST. **a** A diagram illustrating the cytoarchitecture of dorsal BNST subnuclei, on the right side of a coronal section of the anterior division of the BNST. An enlarged illustration on the right-hand side depicts the dorsolateral BNST in more detail. al, anterolateral BNST; ov, oval nucleus of the BNST; ad, anterodorsal BNST; LV, lateral ventricle; AC, anterior commissure; ic, internal capsule. **b** Comparison of Venus-expressing neurons in the dorsolateral BNST between gonadally intact- and gonadectomized mice. Venus-expressing neurons are stained with immunohistochemistry (brown-colored), and subsequently, Nissl staining was carried out using the same section to identify the subnuclei of the BNST. Venus-expressing neurons were stained more intensely not only in the soma but also in the fibers in proestrous female mice compared with intact males. AC, anterior commissure; ic, internal capsule; LV, lateral ventricle. Scale bar = 200 μm

GDX was carried out in both male and female mice at 3 months of age to examine whether the sex differences in the dorsolateral BNST are affected by gonadal steroids. The diagram in Fig. 4a illustrates the cytoarchitecture within the dorsolateral BNST that comprises alBNST and ovBNST. As shown in Fig. 4b, the number of Venus-expressing neurons decreased markedly in ovariectomized females in comparison with proestrous females. In contrast, the number of Venus-expressing neurons increased markedly in the dorsolateral BNST in orchiectomized males compared with gonadally intact males.

**Fig. 5:**
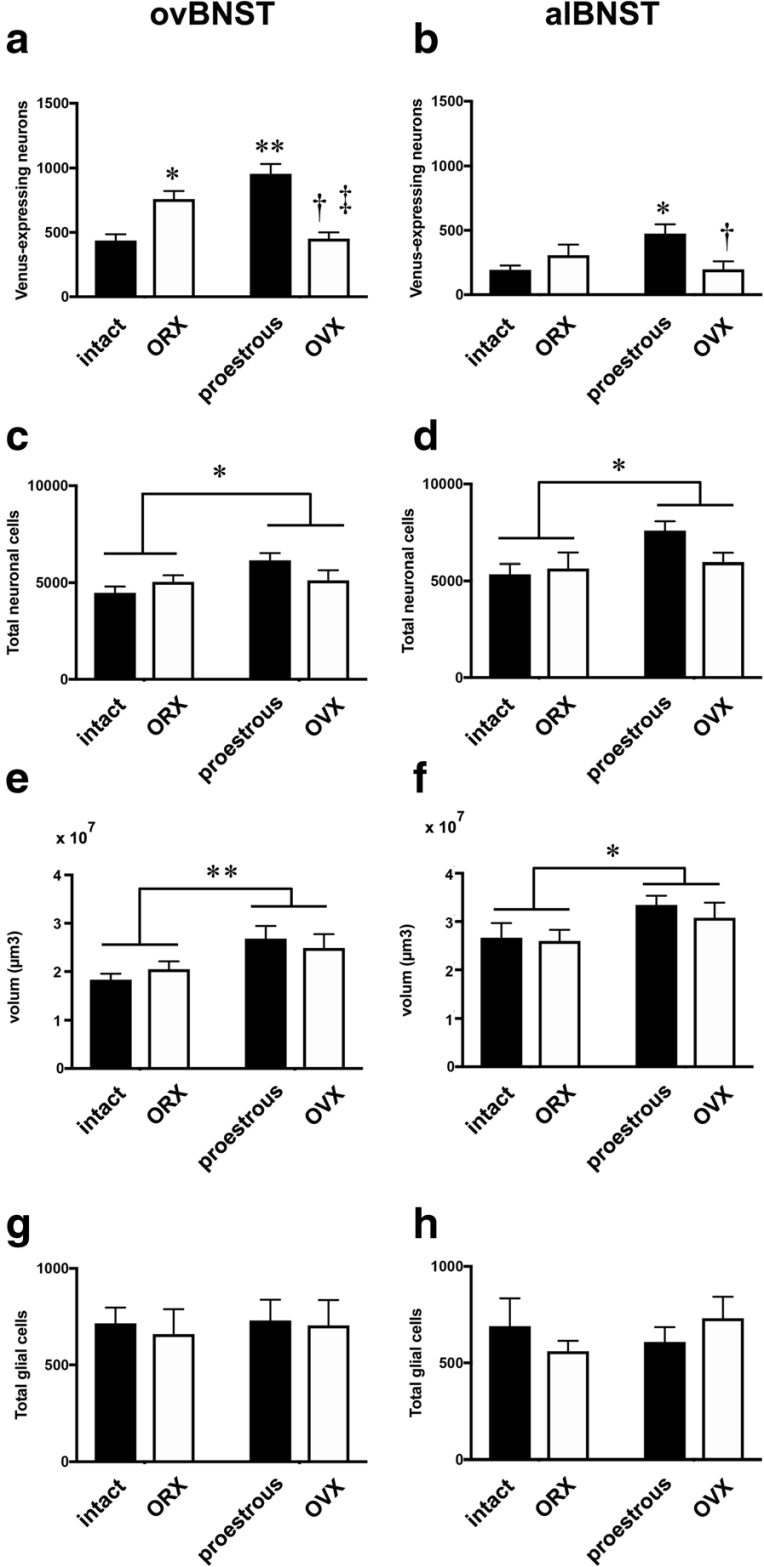
Effects of sex or gonadectomy on the morphological features of Venus-expressing neurons in alBNST or ovBNST. Effects of sex or GDX on the number of Venus-expressing neurons in ovBNST (**a**) or alBNST (**b**). Groups of Sham-orchiectomized males (intact), orchiectomized males (ORX), Sham-ovariectomized proestrous females (proestrous), and ovariectomized females (OVX) were analyzed by two-way ANOVA. There was a significant interaction between sex and GDX in both subnuclei. Multiple comparisons test indicated female predominance in the number of Venus-expressing neurons in either ovBNST or alBNST. ovBNST: *p < 0.01 vs. intact males, **p < 0.0001 vs. intact males,  ^†^*p* < 0.0001 vs. proestrous females, ^‡^*p* < 0.01 vs. ORX. alBNST: *p < 0.05 vs. intact males, ^†^*p* < 0.05 vs. proestrous females. Effects of sex or GDX on the total number of neurons in ovBNST (**c**) or alBNST (**d**). As regards the total number of neurons, there was a significant main effect of sex in either ovBNST (**c**) or alBNST (**d**), but there was no interaction between sex and GDX, nor was these a main effect of GDX. *, *p* < 0.05. Effects of sex or GDX on the volume of ovBNST (**e**) or alBNST (**f**). There was a significant main effect of sex on the volume of the nucleus in either ovBNST (**e**) or alBNST (**f**), but there was no an interaction between sex and GDX, nor was these a main effect of GDX. **, *p* < 0.01, *, *p* < 0.05. Effects of sex or GDX on the number of glia in ovBNST (**g**) or alBNST (**h**). Neither an interaction between sex and GDX nor a significant main effect of sex or GDX was observed for the number of glial cells in either ovBNST (**g**) or alBNST (**h**)

We next analyzed the data in alBNST and ovBNST, using the stereological method, to estimate the number of Venus-expressing neurons, as well as total number of neurons, in each subnucleus, the volume of each subnucleus, and the number of glial cells in respective subnuclus, in four groups of animals, i.e., gonadally intact males, orchiectomized males, proestrous females, and ovariectomized females. In ovBNST, two-way ANOVA indicated a significant interaction, for the number of Venus-expressing neurons, between sex and GDX (*F* (1, 27) = 45.81, *p* < 0.0001). Tukey’s multiple comparisons indicated that the number of Venus-expressing neurons was significantly greater in proestrous females vs. intact males (*p* < 0.0001), significantly greater in proestrous females vs. ovariectomized females (*p* < 0.0001), and significantly greater in orchiectomized males vs. intact males (*p* < 0.01) (Fig. 5a). Unexpectedly, the number of Venus-expressing neurons was greater significantly in orchiectomized males vs. ovariectomized females (*p* < 0.01) (Fig. 5a). In alBNST, two-way ANOVA also indicated a significant interaction between sex and GDX (*F* (1, 27) = 8.943, *p* < 0.01). Tukey’s multiple comparisons test indicated that the number of Venus-expressing neurons was significantly greater in proestrous females vs. intact males (*p* < 0.05) and significantly greater in proestrous females vs. ovariectomized females (*p* < 0.05) (Fig. 5b). There was no significant difference, in the number of Venus-expressing neurons, between intact males and ovariectomized females, or between orchiectomized males and proestrous females, in either ovBNST or alBNST (Fig. 5b).

We also analyzed the number of Venus expressing neurons in intact male and proestrous female mice or gonadectomized male and female mice, with respect to the effects of location and sex (not shown in Figures). For intact males and proestrous females, two-way ANOVA indicated a significant main effect of sex (more predominant in females than in males) (F (1, 28) = 44.37, *p* < 0.0001) and a significant main effect of location (more predominant in ovBNST than in alBNST) (F (1, 28) = 36.34, *p* < 0.0001), but there was no interaction between location and sex. There were also significant main effects of sex (more predominant in females) and location (more predominant in ovBNST), without an interaction between sex and location, in gonadectomized mice (data not shown).

Next, the effect of sex or GDX on the total number of neurons was analyzed in the dorsolateral BNST subnuclei. Two-way ANOVA indicated a significant main effect of sex in either ovBNST (F (1, 27) = 5.021, p < 0.05) or alBNST (F (1, 27) = 4.519, *p* < 0.05). However, there was no interaction between sex and GDX in either ovBNST or alBNST, nor was there a main effect of GDX in either ovBNST (F (1, 27) = 0.3381, *p* = 0.57) or alBNST (F (1, 27) = 1.204, *p* = 0.28). An analysis was made for the effect of sex or GDX on the volumes of dorsolateral BNST subnuclei. Two-way ANOVA indicated a significant main effect of sex in either ovBNST (F (1, 28) = 9.268, p < 0.01) or alBNST (F (1, 27) = 4.903, *p* < 0.05). However, there was no interaction between sex and GDX in either ovBNST or alBNST, nor was there a main effect of GDX in either ovBNST (F (1, 28) = 0.003, *p* = 0.96) or alBNST (F (1, 27) = 0.3989, *p* = 0.53).There was no effect of sex or GDX on the number of glial cells in either alBNST or ovBNST (Fig. 5g, h).

## Discussion

We showed for the first time that CRF neurons in the two BNST subnuclei of the mouse, i.e., ovBNST and alBNST, show a female-biased sexual dimorphism using a mouse line in which Venus is driven by the *Crf *promotor. The present results are consistent with previous reports in the rat [14, 15], reinforcing the female predominance in the number of BNST CRF neurons across species. CRF neurons that were identified by Venus expression are mostly confined to the anterior division of the BNST, consistent with the previous reports in the rat [6, 10]. We showed further that they constitute a distinct population from the principal nucleus of the BNST, which is a well-known male-biased sexual dimorphic nucleus [24–26]. In the present study, the number of Venus-expressing neurons was greater in ovBNST compared with alBNST, recapitulating the previous observations in the rat that CRF neurons are expressed most prominently in ovBNST [6, 11].

Remarkably, the number of Venus-expressing neurons changed markedly by the elimination of sex steroids by GDX in adult male and female mice. In females, the number of Venus-expressing neurons decreased significantly in ovBNST and alBNST following OVX. In males, the number of Venus-expressing neurons increased in these subnuclei following ORX, although the changes were statistically significant only in the ovBNST. Thus, GDX brought about a change in the number of Venus-expressing neurons in an opposite direction between males and females. It is unlikely that an absence of gonadal steroids in adulthood elicits either apoptosis or mitotic division of CRF neurons, so the apparent changes in the number of Venus-expressing neurons, observed in the present study, were considered to have resulted from the changes in expression levels of cytoplasmic Venus content which was dependent on the CRF promoter activity. This idea was reinforced by an observation that the total number of neurons and the volume of the nucleus were not affected significantly by GDX. Unexpectedly, the number of Venus-expressing neurons in the orchiectomized male group was significantly greater than that in the ovariectomized female group. It is not clear whether this ‘reverse effect’ of GDX is based on the genetic- or epigenetic difference (dependent on perinatal gonadal hormone levels) between the sexes.

It was also shown in the present study that the number of Venus-expressing neurons in the dorsolateral BNST increased markedly during the course of early development till adulthood. In the mouse BNST, neurons had already been postmitotic by embryonic day 15.5 [27]. Maturation of BNST is then accomplished by apoptotic cell death that reaches the peak at P5, and the number of neurons stabilizes thereafter [28]. Therefore, the postnatal increase in the number of Venus-expressing neurons is unrelated to the mitotic increment of CRF neurons, and it must have resulted from the activation of *Crf* promoter during development. The *Crf* mRNA expression was also markedly increased postnatally in the hypothalamus and the amygdala in rodents [29, 30], so the increase in CRF expression during development may not be specific for the BNST.

Since both alpha and beta subtypes of the estrogen receptor are distributed across dorsolateral BNST [31], estrogen is capable of affecting CRF neurons located in the region. Multiple estrogen response element half-sites are present upstream of the *Crf* open-reading frame [32]. *Crf* promoter activity may be activated by estrogen since *Crf* gene expression was decreased by OVX in the mouse PVH, and treatment of ovariectomized females with estradiol led to a recovery of *Crf* expression within 12 h [33]. Androgen receptors are also distributed throughout the dorsolateral BNST [34], and an androgen response element-like sequence is present upstream of the *Crf* open-reading frame [35]. Therefore, testosterone may possibly suppress *Crf* promoter activity directly. It was reported in the rat that the exposure to testosterone at an early postnatal stage abolishes the female-biased sexual dimorphism in the BNST [15], suggesting a role of androgen in the sex-dependent differentiation of the BNST. Since estrogen can be produced by conversion of testosterone by tissue aromatase, it is not clear whether androgen exerts its effect directly via androgen receptors, or whether estrogen is responsible, in the regulation of transcription process, for inducing the sexual dimorphism of the BNST [36].

In addition to the sex difference in the number of Venus-expressing neurons, the total number of neurons was also significantly greater in females than in males in either ovBNST or alBNST, yet the number of neurons was unaffected by the absence of gonadal steroids in either sex in adulthood. This is consistent with the previous report in the rat that the number of neurons was greater in females in the anterior region of the lateral division of BNST [37], which is identical to the ovBNST in the present nomenclature. Interestingly, the number of neurons in this subnucleus was greater in 3-month-old male rats that underwent ORX on the day of birth than in control males [37]. Conversely, the number of neurons was smaller in females which had been injected with testosterone on the day of birth compared with control females [37]. Therefore, female-biased sexual dimorphism in this nucleus was dependent on gonadal steroid levels at an early developmental stage in the rat; we assume that the female predominance in the total number of neurons in the mouse ovBNST and alBNST, observed in the present study, may also be related to the early postnatal exposure to gonadal steroids. The female-biased sexual dimorphism of the dorsolateral BNST makes a contrast to the well-recognized male-biased sexual dimorphism of the principal nucleus which is located just rostral to the dorsolateral BNST.

The dorsolateral BNST is one of the relay centers for integrating emotional responses including anxiety and fear [12, 38]; it receives neural projections from the cortical, limbic, and brain stem regions and projects directly to the hypothalamic and midbrain/brainstem regions, which give rise to emotional or autonomic outflows [39–41]. Although the BNST is a composite structure containing multiple neuronal species, CRF neurons in the BNST are specifically implicated in stress- and anxiety-related behaviors in this nucleus [42]. Recently, it was reported that selective activation of ovBNST, by optogenetic methods, elicits anxiogenic responses [41, 43], reinforcing the hypothesis that the CRF neurons in ovBNST may be involved in the processing of anxiety-like behavior.

It is well-documented that anxiety-related behaviors are dependent on the levels of gonadal steroids in adulthood; for example, GDX is associated with increased anxiety in adult male rats [44, 45], and this can be reversed by testosterone supplementation [44, 46, 47]. However, it is not clear what brain region(s) is responsible for the gonadal steroid-dependent anxiety-like behavior. Since expression of CRF in the dorsolateral BNST is regulated robustly by gonadal steroid, it is tempting to speculate that the CRF neurons in the dorsolateral BNST may be related in part to the sex difference in fear and/or anxiety responses under stress, as well as related pathological conditions, such as anxiety disorders or depression [48–51].

To understand the sexual difference in brain function, it is imperative to understand the morphological differences between the male and female brain in detail, and sexually dimorphic nuclei are the best targets to explore in the first place. Thus, a morphological study of sex differences in the BNST is key for revealing the mechanisms of local handling of emotional information such as fear and anxiety, and in this context, further studies are required to unravel the functional significance of the female-biased expression of CRF neurons in dorsolateral BNST.

## Conclusion

Our findings indicated that Venus-expressing CRF neurons present a female-biased sexual dimorphism in ovBNST and alBNST, two subnuclei in the dorsolateral BNST. The absence of gonadal steroids in adulthood abolished the female-biased sex difference in the number of Venus-expressing neurons. Thus, the *Crf* promoter activity in the dorsolateral BNST was highly dependent on gonadal steroids. CRF neurons expressed in the BNST are implicated in the anxiety-like behavior, and we speculate that the female-biased expression of CRF in the dorsolateral BNST may possibly be related to the sex differences in the processing of emotional responses including anxiety.

## Abbreviations

alBNST: Anterolateral BNST
ANOVA: Analysis of variance
BNST: Bed nucleus of the stria terminalis
CeA: Central nucleus of the amygdala
CRF: Corticotropin-releasing factor
GABA: Gamma-aminobutyric acid
GDX: Gonadectomy
GFP: Green fluorescent protein
ovBNST: Oval nucleus of the BNST
OVX: Ovariectomy
P2: Postnatal day 2
P5: Postnatal day 5
PVH: Paraventricular nucleus of the hypothalamus
Venus: Modified yellow fluorescent protein
