# Correction to: Female-biased sexual dimorphism of corticotropin-releasing factor neurons in the bed nucleus of the stria terminalis

**DOI:** 10.1186/s13293-019-0224-z

**Published:** 2019-02-19

**Authors:** Katsuya Uchida, Hiroko Otsuka, Masahiro Morishita, Shinji Tsukahara, Tatsuya Sato, Kenji Sakimura, Keiichi Itoi

**Affiliations:** 1 Laboratory of Information Biology, Graduate School of Information Sciences, Tohoku University, Sendai City, Japan; 2 Department of Regulation Biology, Graduate School of Science and Engineering, Saitama University, Saitama City, Japan; 3 Department of Cellular Neurobiology, Brain Research Institute, Niigata University, Niigata City, Japan

**Correction to: Biol Sex Differ**

**https://doi.org/10.1186/s13293-019-0221-2**

Following publication of the original article [1], we noticed a number of errors. The errors and corrections are listed below, with corrections indicated in **bold font**. The errors do not impact the conclusions of the paper. We, the authors, apologize for any inconvenience this may have caused.

1. Under the heading *Morphometrical analysis by stereology*, the sentence
  “These parameters were examined in gonadally intact males (Sham-orchiectomized), orchiectomized males, proestrous females (Shan-ovarictomized), or ovariectomized females” should read as follows:
  “These parameters were examined in gonadally intact males (**sham**-orchiectomized), orchiectomized males, proestrous females (**sham**-ovarictomized), or ovariectomized females.”

2. Under the heading *Morphometrical analysis by stereology*, the sentence
  “We first traced the outlines of ovBNST and alBNST on the left side of each section to calculate the volume of the selected area and the number of cells within the area” should read as follows:
  “**We traced the outlines** of ovBNST and alBNST on the left side of each section to calculate the volume of the selected area and the number of cells within the area.”
3. The caption to Figure 5, third sentence
  “Groups of Sham-orchiectomized males (intact), orchiectomized males (ORX), Sham-ovariectomized proestrous females (proestrous), and ovariectomized females (OVX) were analyzed by two-way ANOVA” should read:
  “Groups of **sham**-orchiectomized males (intact), orchiectomized males (ORX), **sham**-ovariectomized proestrous females (proestrous), and ovariectomized females (OVX) were analyzed by two-way ANOVA.”
